# Preventive Care and Outcomes of Interprofessional Medicare Annual Wellness Visits for Older Adults

**DOI:** 10.3390/ijerph192416752

**Published:** 2022-12-13

**Authors:** Camlyn Masuda, Lovedhi Aggarwal, Michele Lani Bray, Christabel K. Cheung, Bum Jung Kim, Aida Wen, Lauren Okamoto, Matthew Uechi, Yeonjung Jane Lee, Kamal Masaki

**Affiliations:** 1 Department of Pharmacy Practice, Daniel K. Inouye College of Pharmacy, University of Hawai‘i at Hilo, Hilo, HI 96720, USA; 2 Department of Family Medicine and Community Health, John A. Burns School of Medicine, University of Hawai‘i at Mānoa, Aiea, HI 96701, USA; 3 Nancy Atmospera-Walch School of Nursing, University of Hawai‘i at Mānoa; Honolulu, HI 96822, USA; 4 Thompson School of Social Work & Public Health, University of Hawai‘i at Mānoa, Honolulu, HI 96822, USA; 5 Department of Geriatric Medicine, John A. Burns School of Medicine, University of Hawai‘i at Mānoa, Honolulu, HI 96817, USA

**Keywords:** interprofessional/interdisciplinary team, geriatric, primary care, interprofessional education (IPE)

## Abstract

As we anticipate a growing population of older adults, we will see an increase in chronic conditions such as dementia and falls. To meet these public health needs, we must systematically provide screening, education, preventive care, and supportive care for older patients and their caregivers in a primary care setting. This will require a workforce trained in providing for the complex medical and psychosocial needs of an older adult population in an interprofessional and collaborative fashion. By integrating geriatric screening tools into an interdisciplinary Annual Wellness Visit teaching clinic, we were able to successfully improve rates of geriatric screening for dementia, depression, falls, medication reconciliation and advance care planning. We also saw improvements in patient care and satisfaction and provided the opportunity for interprofessional collaboration and education for students in medicine, nursing, pharmacy and social work.

## 1. Introduction

There is a significant shift in age demographics occurring in America and worldwide. As of 2020, there were 54.1 million adults ages 65 years and older in the United States (U.S.), which accounts for 16% of the total population. It is projected that by 2060, the nation’s population of adults 65 years and older will make up 23%, or nearly one-quarter of the population [1].

Since Hawai‘i’s residents boast an overall life expectancy that is the highest in the U.S., the island state is expected to be on a faster timeline in experiencing the challenges of their burgeoning older adult population. It is estimated that by 2040, the proportion of adults ages 60 years and older in the state of Hawai‘i will reach 28.5% [2,3], which is more than one-third greater than the projection for the entire United States. Thus, improving care and preventing chronic conditions and hospitalizations in the geriatric population in Hawai‘i would serve as a model for the rest of the U.S. and other countries.

The aging patient population presents a public health challenge for health care providers, as we expect to see an increase in falls, dementia, mental health conditions such as depression, and functional decline. In the environment of workforce shortages for geriatricians [4,5] and primary care providers [6], allied health professionals must be trained on geriatric interventions; thereby, making positive contributions to the clinical team in the screening, preventive care including medication reconciliation, advance care planning, and management of chronic conditions and vulnerabilities for this older population. 

Falls among adults over the age of 65 years represent a significant public health concern. Each year, approximately one out of four people ages 65 years and older fall, and falls account for 2.8 million emergency admissions of older adults annually [7]. About 95% of hip fractures are due to falls, with hip fracture survivors experiencing significantly worse mobility, independence in function, health, quality of life, and higher rates of institutionalization than age matched controls [8]. Over the past decade, there has also been a concerning increase in fall-related traumatic brain injuries among older adults, which are associated with high rates of morbidity and mortality [9]. Moreover, the cost of falls to society is great - the total medical cost of falls totaled more than $50 billion in 2015 in the United States, and Medicare and Medicaid, federal and state government funded medical insurance, shouldered 75% of these expenditures [10]. When risk factors are adequately addressed, older adult falls are largely preventable [7]. Specifically, risk factors for falls include age, medication, chronic disease conditions, vision loss, lower body weakness, problems with gait and balance, feeling sad or depressed, and home hazards [11,12]. 

Alzheimer’s Disease and Related Dementias (ADRD) are another important public health concern. Dementia is the sixth leading cause of death in the U.S. [13]. Currently, 5.8 million Americans live with dementia. However, this number is projected to rise to nearly 14 million by 2060 [14]. Unfortunately, these numbers likely underestimate how many people are actually living with dementia due to the lack of early diagnosis/detection and underreporting.

The risk for depression in older adults is higher than the adult population as depression is more common among those who have other medical conditions or limited function [15]. Regular screening for depression in older adults is vital to identify those in need and approach ways to treat depression, since less than half of older adults with depression would get help from a health professional [16]. 

Falls, dementia and depression ultimately impact the ability of older adults to care for themselves, manage other chronic conditions, and continue living independently. To meet the multifaceted nature of these public health needs, we must systematically provide screening, education, preventive care, and supportive care for older patients. Completing an accurate medication reconciliation at every visit is an important preventive care strategy as it decreases medication discrepancies and errors [17]. Another important aspect of preventive care is advance care planning (ACP) which helps to ensure patients’ wishes for care are known in case they are unable to make decisions for themselves, and may prevent unwanted life extending procedures [18]. ACP in older adults improves patient satisfaction and reduces stress, anxiety and depression for family members [19]. Utilization of trained non-physician healthcare professionals (nurse or allied health worker) in patient centered discussions on ACP improved completion [19]. An effective clinical practice requires a multi-disciplinary geriatrics workforce trained to assess and intervene upon the complex medical and psychosocial needs of an older adult population in an interprofessional and collaborative fashion in a primary care setting. 

Recognizing these needs, the United States government organization, the Health Resources and Services Administration (HRSA) created the Geriatrics Workforce Enhancement Program (GWEP). In 2015, the University of Hawai‘i John A. Burns School of Medicine was one of 44 nationwide recipients of the GWEP grant (the Pacific Islands GWEP). The main objectives of this grant were to integrate geriatrics into primary care clinical sites and include interprofessional training. The Pacific Islands GWEP took advantage of current payment delivery reforms and quality reporting measures as an opportunity to make falls prevention and dementia screening part of routine clinical primary care practice. With the passage of the Affordable Care Act (ACA) in 2010, the Center for Medicare and Medicaid Services (CMS) began covering many preventive care services and providing a free Annual Wellness Visit (AWV) to beneficiaries of Medicare (elderly or disabled) [20]. The AWV is a yearly appointment with the primary care provider to create or update a personalized prevention plan based on a patient’s health and risk factors. It typically includes more general preventive screening for cancers, low bone mass, cardiovascular disease and diabetes and includes alcohol education, tobacco cessation and immunizations. Other screening options that were more geriatric-focused could also be done. Geriatric syndrome focused screening was incorporated into the AWV for a family medicine primary care clinic using an interprofessional team (IPT) approach. The unique aspect of the intervention in this study was having faculty from local schools/colleges of medicine with geriatrics specialization, pharmacy, nursing and social work, that provided efficiency, expertise, and an opportunity to enhance training for health provider trainees/students in addressing the needs of older adults, and in interprofessional practice.

## 2. Materials and Methods

### 2.1. Participants

The intervention site was a family medicine clinic in Central O‘ahu, a rural part of Hawai‘i. Patients eligible for an AWV were identified from a list of patients who had Medicare insurance coverage. Patients were contacted from this list and were scheduled if they were available and interested in team care management. Patients that declined or were unable to come in on the available dates for the IPT AWV were offered the opportunity to have an AWV with their primary care provider.

During each clinic, faculty and one or two students from each discipline (nursing, social work, pharmacy) participated. Faculty from family medicine and geriatrics also participated. Although the AWV with the interdisciplinary team continues, this article includes data from *n* = 31 patient AWVs between February 2016 and April 2018. 

### 2.2. Intervention

Patient service representatives (PSR) contacted potential participants to inform them about the IDT AWV, which included providers and students from different disciplines to introduce patients to team-based care. Upon confirming patients’ verbal consent, the PSR scheduled the AWVs into the half-day interprofessional clinics that occurred once every one to two months during the academic year (September–March).

**Figure 1:**
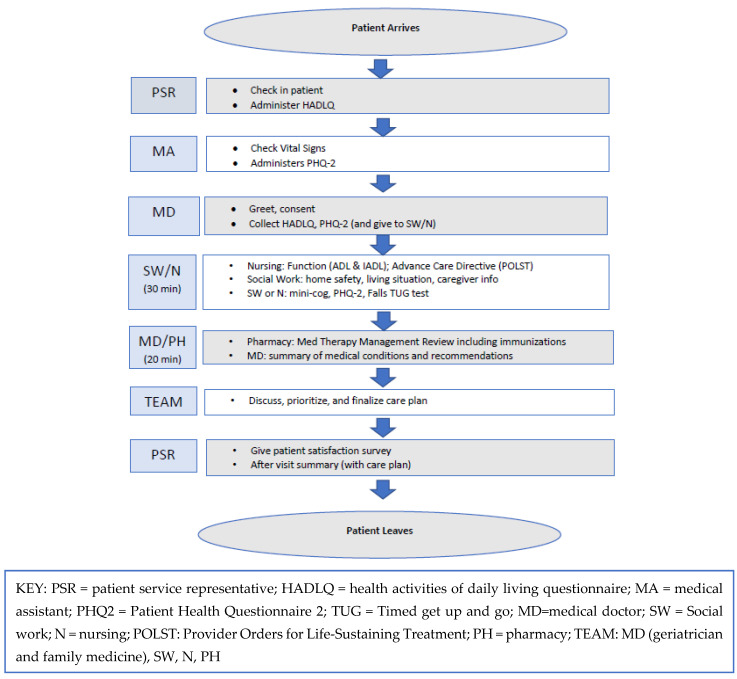
Annual wellness visit clinic workflow.

Between one to three patients were scheduled per half-day clinic. The GWEP team arrived 30–60 min prior to the first patient’s AWV appointment to review the patient’s chart together and prepare students for the visit. Each patient’s AWV involved one supervising physician, one to two students from each discipline (nursing, pharmacy, and social work), and one supervising preceptor for each discipline. Prior to the visit, the students and preceptors discussed specific issues relevant to their respective disciplines that were found in the chart review. The workflow and tasks are summarized in Figure 1. 

Once the patient arrived, the PSR checked them in. The medical assistant (MA) then took the patient’s vital signs and brought them to an exam room. The patients were also asked to complete a health activity of daily living questionnaire (HADLQ) and Patient Health Questionnaire 2 (PHQ-2) which are tools used to assess the patient’s functional ability and risk for depression, respectively [21,22]. The rates of completion were not collected or reported in this manuscript however, they are reported in the methods section since they were performed during the visit and meet the required components of the health risk assessment required by Medicare [23]. If the patient was not able to complete these on their own, the MA aided.

The physician then saw the patient briefly to greet them and obtain consent for team care. The physician collected the HADLQ and gave it to the Nursing (N) and Social Work (SW) students. Together, the nursing and social work students interviewed the patient first for about 20–30 min, which included reviewing the HADLQ, administering the Mini-Cog exam (screening tool for dementia), and discussion of advance care directives and/or Provider Orders for Life-Sustaining Treatment (POLST). Social work students assessed safety at home, living and financial situation, and assessed the need for a caregiver. Nursing students also performed the Timed Up and Go (TUG) test recommended by the Centers for Disease Control [24], to assess mobility if the patient had any recent falls or was at risk for falls, and determined causes of the fall if it occurred. Upon completion of their visit, they would provide a brief summary (1–3 min) of their assessments to the pharmacy (PH) student and physician, who would then interview the patient next for about 20 min. The PH students would perform medication reconciliation with the patient, including confirming any over-the-counter medications and supplements, and verifying immunizations completed. After the PH student and physician saw the patient, a brief summary (1–3 min) of their assessments was given to the entire GWEP team. 

At the end of the visit, the physician summarized a preliminary plan for the patient, and addressed any urgent issues, if any. The patient then received a printed after visit summary sheet that included the preliminary care plan and was informed to follow up with their primary care physician. Lastly, in closing the visit, the patients were asked to complete an optional, anonymous patient satisfaction survey that they returned to the PSRs. Meanwhile, the GWEP Team met to discuss the patient’s treatment plan and completed a Geriatric Interdisciplinary Care Summary (GICS) worksheet. This is a useful framework for developing a comprehensive geriatric care plan organized around eight domains: rehabilitation, cognitive, emotional, medical/surgical, nutritional, environmental, social/caregiver, and economic [25]. The team worked together to address challenges and contributing factors to the patient’s medical issues. As the team prioritized the patient’s issues and developed a final care plan, the supervising physician completed documentation in the Electronic Medical Record (EMR) and forwarded the care plan to the patient’s primary care physician (PCP) for follow up. The supervising physician and PCP work together in the same clinic, so the PCP was able to access the forwarded care plan within the EMR.

The students completed surveys to assess their experience working in the GWEP team, and students who attended more than one AWV wrote a reflection statement. Results from these statements are not included in this article and will be published in another manuscript focusing on the student’s perspectives. The total time spent per patient was 1 h, and the total time per clinic was about 3 h.

The Institutional Review Board at the University of Hawai‘i approved this project (approval number 2021-00840) as non-human research. Informed consent from the participants was not needed as this was a quality improvement project.

### 2.3. Data Analysis

Descriptive statistics were used to characterize patient demographics and patient satisfaction scores. Means and standard deviations were used for continuous variables, and percentages for categorical variables. Screening rates for each geriatric syndrome were calculated for the patients before and after participating in the AWV, looking particularly at dementia, depression, falls, medication reconciliation, and advance care planning. Manual chart reviews by one of the authors was performed to collect screening rates prior to the IPT AWV and after the visit. Data were collected from prior office visit notes (medication reconciliation) or the search feature within the chart for completion of the screening tools (dementia, falls, depression) and if advance care planning was completed. Differences in rates before and after participation in the AWV were analyzed by using paired *t*-tests. 

## 3. Results

### 3.1. Demographics

We have complete data from 31 patients who enrolled in the interdisciplinary AWV over two years (2016–2018). Patients’ mean age was 72.16 (SD = 6.73) years (range = 63–89 years), 74.2% were female, and the ethnic distribution was 16.1% White, 29.0% Asian, 32.3% Hawai‘ian/Pacific Islander, and 22.6% Other/Mixed/Unknown. 

### 3.2. Patient Outcomes

With the interprofessional team approach for the AWV, we found significant increases (*p* < 0.05) in screening rates for dementia, depression, falls, performance of medication reconciliation and advance care planning (Table 1) after the IPT AWV was performed compared to prior to the intervention. A subgroup analysis stratified by age found that improvements in screening rates were higher among those ≥75 years old for dementia, depression and falls when compared with those <75 years old. In contrast, improvement in advance healthcare planning was greater among those <75 years old. There was no difference in improvement of medication reconciliation between the 2 age groups (Table 2).

### 3.3. Patient Satisfaction

A total of 23 patients returned the patient satisfaction survey (Table 3). All (100%) were generally satisfied with their care; 95.7% felt their needs were taken care of at the visit; and 78.3% would like to participate in team care for future visits. One question about learning new things was answered by 11 patients only, with 90.9% saying they learned new things. 

## 4. Discussion

During the past ten years, the U.S. healthcare system has been undergoing several reforms. One is the shift towards more preventive care, including providing a free AWV to beneficiaries of Medicare [20]. AWVs are reimbursed separately from regular office visits, which provides an additional source of revenue to support participation from other healthcare team members. The CMS guidelines for AWVs state that other healthcare professionals, such as nurse practitioners, physician assistants and pharmacists, are able to perform the service [23,26]. 

This reflects another significant shift—recognition of the growing importance of interprofessional practice. Some practices have incorporated team based AWVs utilizing physicians, nurses or pharmacists and found improved utilization of preventive services [27,28]. For example, the inclusion of pharmacists enables the provision of comprehensive medication management services and updating vaccines for senior clinic patients [28,29]. Consequently, to build interprofessional collaborative skills into our healthcare workforce, interprofessional education (IPE) has become a required part of curriculum accreditation for many health professions schools, such as pharmacy, nursing, social work, and medicine, in the last several years [30,31,32,33]. 

Prior to this project in 2014, the family medicine clinic partnered with the state’s only college of pharmacy to have a pharmacy faculty and students assist physicians in medication management. Building upon this legacy, this project expanded and improved upon comprehensive preventive care by integrating students from the schools of nursing and social work; meanwhile, providing geriatrics and interprofessional training at a primary care clinical site through the AWV mechanism.

There are few studies focusing on IPE and AWVs. However, our findings were consistent with these preceding pilot studies described in the literature, whether with students or with practicing pharmacists or nurses [27,28,29,34]. In particular, our findings were most similar to Zorek et al.’s study that described a family medicine clinic inclusive of pharmacy, nursing, and physician trainees, that provided general preventive care (i.e., cancer screening, vaccines, smoking and alcohol) for 34 Medicare beneficiaries ages 66–74 years [28]. AWVs were shown to be effective in promoting the use of general preventive services with statistically significant improvements in pneumococcal vaccines, mammograms, fecal occult blood testing, and dual x-ray absorptiometry osteoporosis screening [20]. In contrast, we focused on the screening of specific geriatric syndromes, and overall achieved statistically significant increases in screening for dementia, depression, falls, medication reconciliation, and advance care planning. In addition, our study also examined adults ≥75 years old. In the subgroup analysis of those ≥75 years old, we found the biggest differences were for screening for falls and dementia. This is appropriate, because older patients are at greater risk for these types of events. There was no difference between genders (data not shown), which was also appropriate since screenings should be performed regardless of gender. By using the IPT to perform the AWV, the clinic observed significant increases in screening rates for dementia, depression, and falls resulting in post screening rates as high as 90%. Because pharmacy students were previously involved in medication reconciliation, changes in medication reconciliation rates were smaller due to a ceiling effect. Specifically, rates were already high at 83.9%, but they did increase medication reconciliation services to 100% of their patients. 

Another area of success achieved is in advance care planning (ACP). According to a 2017 study by Yadav et al., only about 46% of adults completed an advance directive [35]. Blackwood et al. (2019) explored barriers to ACP as perceived by nurses and other healthcare professionals and found that the two most important barriers were lack of education and insufficient time [36]. Thus, ACP education for trainees in the context of an AWV clinic would be an ideal way for them to practice these skills. This was done as a small quality improvement project in an internal medicine residency program. While it did demonstrate feasibility, evaluations were focused on residents’ skills and perspectives, rather than increased rates of ACP. Additionally, other disciplines were not included [37]. In our study, students participated in a brief didactic session on ACP, and were given time to discuss ACP in the context of holistic and preventive care. This resulted in an impressive increase in ACP completion, from 29% prior to our study to 84% completion afterward. It should also be noted that ACP is an optional part of the AWV, and if performed, qualifies for additional reimbursement by Medicare. 

Another important outcome is patient satisfaction. Overall, patients participating in the AWV reported satisfaction with team care, feeling that their needs were taken care of, and that they were able to learn something from the visit. The majority of patients also indicated interest in participating in future appointments with an IPT, which was an indicator that they were satisfied with the IPT. 

This study had its limitations. One limitation was the small sample size from one clinic in the State of Hawai‘i. Participants who had Medicare were offered the appointment for the AWV and informed that it would be a team-based visit prior to scheduling the appointment and were only offered a limited number of appointment days (once a month) and times. Once the appointment slots were filled no other eligible participants were offered the IPT visit and this may have prevented other eligible people from participating. Another limitation is that there was no randomization of the participants. Additionally, the pre-post design of the same subjects without a comparator group of one organizational model limits its ability to show that the intervention directly led to improvements in screening and generalizability. However, as a pilot study determining possible efficacy, it was an adequate sample. Nonetheless, this study is encouraging, because our results had findings similar to other primary care clinics using a team approach to increase preventive care services through providing AWVs. 

A consideration for future studies to determine efficacy of the team-based care AWV would be to add a comparator group of a similar patient population who received an AWV from clinic physicians and residents. Additionally, obtaining data on current screening rates for the entire population of patients that meet the inclusion criteria would be helpful to compare screening rates with the intervention group. Including additional outcomes such as measuring difference in number of falls will provide an important end point that will improve patients’ health and possibly reduce hospitalizations and healthcare costs. Future studies should increase the frequency of IPT AWV clinic sessions. This strategy would enable greater flexibility for patient scheduling, provide more patients for students to work with, and give students time to engage in follow-up care. The intervention study by Zorek et al. [28], included a follow-up call to patients one month after the clinic visit to follow up on the status of recommended screening tests and assist with scheduling. Providing a follow-up phone call would help ensure that screening tests were performed and determine whether the patient was able to follow up with their primary care provider. Education on specific ACP skills or tools may help increase ACP completion rates. These strategies may improve financial and quality metrics, as more patients receiving AWVs would help meet the goal of percent completion of ACP. Further studies are needed to determine if revenue from the services would adequately fund the IPT and support teaching services.

Although this study was completed in a rural area in Hawai‘i, the methods and intervention may be used in any clinical setting that manages older adults. Clinics may develop partnerships with healthcare colleges and universities to provide the IPT faculty and students. With the increased use of telehealth, the students and faculty may attend via video which will be helpful for clinics in rural settings [38,39]. In countries that do not have government subsidized visits specifically for preventative care, parts of the geriatric screening services provided in this study can be implemented in increments as part of other patient visits. 

Another aspect to consider is increasing the utilization of Annual Wellness Visits. In 2016, only 23% of Medicare patients completed an AWV despite it being a free visit [40]. The improvement in screening rates in this study by performing AWVs with an IPT with students may be used as a way to encourage and advertise to patients to utilize the AWV, as they will have a team of providers in addition to their primary care physician assisting with their care. 

## 5. Conclusions

By integrating geriatric screening tools into an interdisciplinary AWV teaching clinic, we were able to successfully improve rates of geriatric screening, meet quality measure incentives, improve patient care and satisfaction, and provide the opportunity for interprofessional education.

## Figures and Tables

**Table 1:** Difference in screening for geriatric syndromes before and after participating in the annual wellness visit. (*n* = 31 with complete data).

Screening for	Pre	Post	Difference	*p* Value
Dementia	19.4%	87.1%	67.7%	<0.0001
Depression	22.6%	87.1%	64.5%	<0.0001
Falls	32.3%	90.3%	58.1%	<0.0001
Medication Reconciliation	83.9%	100%	16.1%	0.0227
Advance Care Planning	29%	83.9%	54.8%	<0.0001

**Table 2:** Difference in screening for geriatric syndromes before and after participating in the annual wellness visit, stratified by age group. (*n* = 31 with complete data).

Screening for	Age < 75 Years (*n* = 20)	Age > 75 Years (*n* = 11)	*p* Value
Dementia (Pre)	25.0%	9.1%	0.2991
Dementia (Post)	80.0%	100%	0.0421
Dementia (Difference)	55.0%	90.9%	0.0419
Depression (Pre)	25.0%	18.2%	0.6765
Depression (Post)	80.0%	100%	0.0421
Depression (Difference)	55.0%	81.8%	0.1447
Falls (Pre)	40.0%	18.2%	0.2272
Falls (Post)	85.0%	100%	0.0828
Falls (Difference)	45.0%	81.8%	0.0487
Medication Reconciliation (Pre)	85.0%	81.8%	0.8250
Medication Reconciliation (Post)	100%	100%	----
Medication Reconciliation (Difference)	15.0%	18.2%	0.8250
Advance Care Planning (Pre)	20.0%	45.5%	0.1444
Advance Care Planning (Post)	90.0%	72.7%	0.2243
Advance Care Planning (Difference)	70.0%	27.3%	0.0429

**Table 3:** Patient satisfaction scores about interprofessional team care visit.

Questions	% Yes Responses
1. Were you generally satisfied with your care today?	100%
2. Were your care needs taken care of today?	95.7%
3. Did you learn new things?	90.9%
4. Would you like to participate in team care in the future?	78.3%

## Data Availability

Not applicable as all data presented in the article.
